# Compensatory Estrogen Signal Is Capable of DNA Repair in Antiestrogen-Responsive Cancer Cells via Activating Mutations

**DOI:** 10.1155/2020/5418365

**Published:** 2020-07-28

**Authors:** Zsuzsanna Suba

**Affiliations:** National Institute of Oncology, Department of Molecular Pathology, H-1122, Ráth György Str. 7–9, Budapest, Hungary

## Abstract

Cancer cells are embarrassed human cells exhibiting the remnants of same mechanisms for DNA stabilization like patients have in their healthy cells. Antiestrogens target the liganded activation of ERs, which is the principal means of genomic regulation in both patients and their tumors. The artificial blockade of liganded ER activation is an emergency situation promoting strong compensatory actions even in cancer cells. When tumor cells are capable of an appropriate upregulation of ER signaling resulting in DNA repair, a tumor response may be detected. In contrast, when ER signaling is completely inhibited, tumor cells show unrestrained proliferation, and tumor growth may be observed. The laboratory investigations of genomic mechanisms in antiestrogen-responsive and antiestrogen-unresponsive tumor cells have considerably enhanced our knowledge regarding the principal regulatory capacity of estrogen signaling. In antiestrogen-responsive tumor cells, a compensatory increased expression and liganded activation of estrogen receptors (ERs) result in an apoptotic death. Conversely, in antiestrogen resistant tumors exhibiting a complete blockade of liganded ER activation, a compensatory effort for unliganded ER activation is characteristic, conferred by the increased expression and activity of growth factor receptors. However, even extreme unliganded ER activation is incapable of DNA restoration when the liganded ER activation is completely blocked. Researchers mistakenly suspect even today that in tumors growing under antiestrogen treatment, the increased unliganded activation of estrogen receptor via activating mutations is an aggressive survival technique, whilst it is a compensatory effort against the blockade of liganded ER activation. The capacity of liganded ERs for genome modification in emergency states provides possibilities for estrogen/ER use in medical practice including cancer cure.

## 1. Introduction

The role of estrogen hormones in breast carcinogenesis has long been suspected based on the results of menopausal hormone replacement therapy (HRT). The major mechanisms of their carcinogenic effect were recognized via biochemical and genetic investigations [1]. Regretfully, in the majority of HRT studies and laboratory investigations, synthetic estrogens were used taking them for bioidentical hormones, while they are endocrine disruptors inducing toxic complications and cancer via a deregulated activation of estrogen receptors (ERs) [2].

About 70% of newly diagnosed breast tumors express detectable ERs, while the remaining 30% seems to be ER-negative [3]. These observations led to the postulation that in ER-positive breast cancers, estrogen-activated ERs contribute to the initiation and growth of tumors, while the nature of ER-negative ones is quite different, being apparently independent of hormonal impacts [4].

During the fight against breast cancer, the pharmaceutical industry developed antiestrogen compounds. Selective estrogen receptor modulators, such as tamoxifen, and selective estrogen receptor degraders, such as fulvestrant, targeted the inhibition of ligand binding domains (LBDs) of ERs [5]. Aromatase inhibitors (AIs), such as letrozole, were developed for the blockade of estrogen synthesis in breast cancer patients so as to keep circulating estrogen concentrations at a low level [6]. Since the early 1970s, the inhibition of endogenous estrogen signal via antiestrogen treatment has become a standard of care for breast cancer cases [7].

Antiestrogen therapy of patients with advanced breast cancer yielded many difficulties from the onset. Considering the whole population of breast cancer cases, antiestrogen treatment could not surpass the “magic” 30% of tumor response rate, similar to the weaknesses of other endocrine therapies, such as oophorectomy or high-dose synthetic estrogen treatment [8]. In the majority of breast cancer cases (≥70%), tumors were not responsive to antiestrogen therapy exhibiting stagnation or even a rapid growth. In addition, about a half of the targeted ER-positive tumors showed primary resistance to antiestrogen therapy [9]. Moreover, a large proportion of patients showing earlier good tumor responses to endocrine treatment later experienced secondary resistance leading to metastatic disease and fatal outcome [10].

Antiestrogens are administered as a long-term prophylactic treatment as well, after surgical removal of breast tumors, and the relapse rate exhibited a marked reduction [11]. Endocrine treatment was the preferred first-line therapy for advanced breast cancer because of its better tolerability compared to chemotherapy. However, a better outcome was observed among node-positive postmenopausal breast cancer cases after using postoperative short-course chemotherapy combined with prolonged tamoxifen treatment as compared with long-term tamoxifen alone [12]. Chemotherapy is basically indicated for patients with positive axillary lymph node metastasis, and hormone therapy should be added if such patients are ER- and/or progesterone receptor- (PR-) positive. An overview of randomized trials established that among women with ER-positive early breast cancer, 6 months of chemotherapy followed by 5 years of adjuvant tamoxifen treatment approximately halved the breast cancer specific mortality rate throughout the next 15 years [13].

Despite the introduction of long-term prophylactic endocrine therapies, late recurrences and dissemination of breast cancer may appear even more decades after the first diagnosis of tumors [14]. Moreover, in breast cancer cases diagnosed and treated at the earliest stage, tumor recurrence and fatal outcome of the disease may frequently occur suggesting that our therapeutic efforts are not appropriate [15, 16]. In addition to the experienced therapeutic difficulties, long-term antiestrogen treatment may induce toxic side effects, such as arterial and venous thromboembolic events and malignancies, particularly in the endometrium [17].

In the early 2000s, estradiol treatment-induced apoptosis of breast cancer cells was reported as a promising key for restoring the response of tumor cells resistant to either tamoxifen or long-term estrogen deprival (LTED) [18, 19]. These observations suggested new opportunities for the improvement of antiestrogen therapies via amplifications of estrogen-induced apoptosis [20].

There are enormous efforts worldwide to reveal the mechanisms of resistance to endocrine therapy developing in the majority of breast cancer cases [21]. Results of genetic studies on endocrine resistant tumors strongly suggested that an increased expression of tyrosine kinase growth factor receptors and the associated unliganded activation of ERs are to be blamed for the survival and aggressive metastatic biology of tumor cells [22, 23]. In addition, breast cancer cells unresponsive to endocrine therapy exhibit acquired activating mutations on *ESR1* gene driving an increased unliganded activation of ERs presumably as a key for their survival [24–27].

Recently, new therapeutic compounds are developed targeting the signaling of growth factor receptor (GFR) tyrosine kinases so as to silence the extreme unliganded activation of ERs in endocrine resistant tumors [24]. However, the double blockade of liganded and unliganded ER activations yielded the modest or even inverse results on breast cancers [28]. In addition, breast cancer cases could hardly tolerate the severe toxic effects of therapies targeting both ER and GFR signaling [29].

Nowadays, clinicians must balance between the risks and benefits of systemic endocrine therapies. Since new potent and more specific antiestrogens, aromatase inhibitors, and tyrosine kinase inhibitors have been developed for breast cancer care, the new challenges are to select the optimal strategy for a given clinical scenario [30]. These experiences suggest that further insights into underlying mechanisms for growth factor and ER interactions are necessary for the improvement of breast cancer therapy.

In the present work, the principal regulatory capacities of estrogen-activated ERs and the importance of balance between their liganded and unliganded activations are illuminated. Tumors, unresponsive to antiestrogen therapy, do not acquire oncogenic adaptation for their survival, but rather, they strive to compensate the blockade of liganded ER activation via an extreme upregulation of unliganded pathways and activating mutations. In antiestrogen-resistant tumors, a rapid response to estrogen treatment clearly justifies that even exhaustively blocked ERs are capable of reactivation fulfilling their physiological roles, the restoration of DNA stability, and initiating a consequential self-directed death.

## 2. Crucial Role of Estrogens, ERs, and Estrogen-Regulated Genes in Mammalian Health

ERs may act as a hub in the regulatory network at cellular level, accumulating and analyzing all signals arriving from different molecular pathways. In turn, estrogen-activated ERs as transcriptional factors drive the genome wide expression of estrogen-activated genes orchestrating all cellular functions [31]. Alterations in ER activation or in ER-regulated transcriptional processes may induce strong compensatory actions, while an uncompensated deregulation of ER signaling leads to serious chronic diseases including cancer [32].

Estrogen hormones such as estrone (E1), estradiol (E2), and estriol (E3) are synthesized by aromatase enzyme via converting androgens to estrogens. Two estrogen receptor isoforms, ER-alpha and ER-beta, are members of the nuclear receptor superfamily, and they exhibit strong crosstalk and interplay. ER-beta is mainly responsible for cellular enlargement, while the role of ER-alpha is crucial in regulating cell proliferation [33]. Both ER isoforms are mandatory regulators of cellular glucose uptake since both the cell growth and mitotic activity require an appropriate supply of fuel for increased metabolic processes [34, 35]. Failure of estrogen signaling induced by either estrogen deficiency or ER resistance leads to a deepening defect of cellular glucose uptake and consequential serious diseases [36, 37].

ER-alpha and ER-beta proteins are expressed via transcriptional activities on *ESR1* and *ESR2* genes in the nucleus. Estrogen binding on the AF2 domain of ERs ensures a liganded activation enabling ERs for the transcriptional activity, while growth factor receptors and further mediators may activate ERs through the AF1 domain via an unliganded pathway. Activated ERs can induce gene expression through both direct and indirect binding to DNA, in the latter case, via an interaction of another transcription factor protein. Moreover, cell membrane-associated ERs may also confer signaling cascades to estrogen-dependent target genes via a nongenomic pathway [31].

Unliganded ER activation through the AF1 domain may be induced through the mitogen-activated protein kinase (MAPK) or protein kinase B (Akt) pathway providing immense reserve capacities for genomic regulation in an estrogen deficient milieu [38]. Low estrogen concentration endangers the liganded activation of ERs, while a compensatory upregulation of unliganded ERs may transiently save the surveillance of genomic machinery [39]. In embryonic life, the ancient AF1 domain of ERs drives primarily the development and differentiation [25]. In adult men and women, the ligand-dependent AF2 activation of ERs enjoys a conspicuous primacy, while the unliganded activation of ERs via the AF1 domain also has a genome wide function. Experimental studies reveal a strong interplay between liganded and unliganded transcriptional activations of ERs [40].

The transcriptional activity of ERs partially results in the expression of protein coding messenger ribonucleic acids (mRNAs), while the vast majority of RNA transcripts are noncoding RNAs (ncRNAs) [41]. MicroRNAs (miRNAs) are small noncoding transcripts that function in the modification of gene expression and translation via binding to mRNAs at specific binding sites [42]. By contrast, ER-induced long noncoding RNA (lncRNA) transcripts are capable of promoting epigenetic gene modifications via their specific chromatin remodeling activities resulting in mutations on targeted genes [43]. lncRNA transcripts of ERs are in close interplay with genome stabilizer proteins, such as p53 and BRCAs, suggesting a pivotal role of these transcripts in the promotion of DNA protecting mutations [41, 44].

Estrogens are outstanding hormones exhibiting a strong, unique upregulative feedback mechanism with their own receptors [32]. Both low and high estrogen levels drive an increased expression and transcriptional activity of ERs so as to restore or augment ER signaling. In turn, both low and high ER expressions require upregulated estrogen synthesis for the improvement or augmentation of crucial estrogen signaling. Upregulation of estrogen signaling displays a unique dichotomy via DNA stabilization, ensuring a safe proliferative activity or apoptosis for healthy cells, while inducing a programmed death for malignant tumor cells.

### 2.1. Estrogen-Activated ERs Drive DNA Stabilization, Cell Proliferation, and Fuel Supply via Regulatory Circuits

At cellular level, activated ERs are the hubs of signaling networks driving the whole genomic machinery through regulatory circuits [32]. At tissue level, the central adipose tissue is the hub of the signaling network controlling and regulating visceral organs and cardiovascular structures through its estrogen synthesis and estrogen receptor activation [45]. Molecular players of all cellular mechanisms are recruited into regulatory circuits receiving their activating signals from ERs and, in turn, sending their signaling reports back to ERs.

### 2.2. DNA Stabilizer Circuit Regulated by Estrogen-Activated ER-Alpha

**Figure 1:**
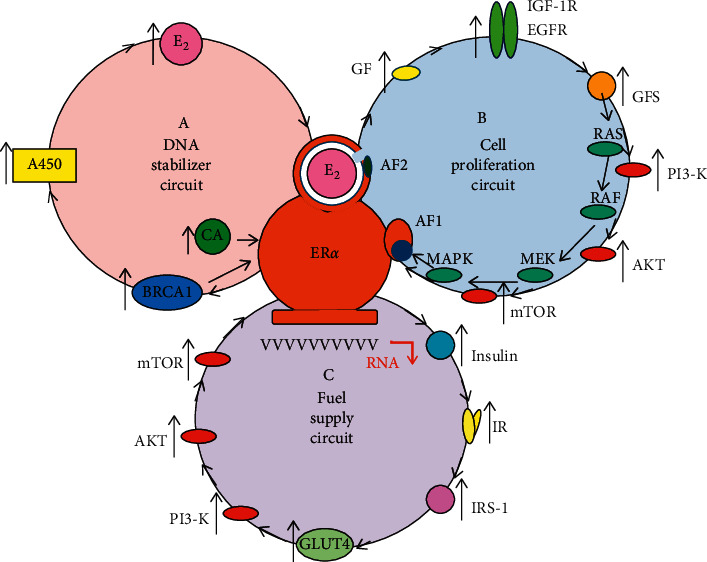
Regulatory circuits driven by liganded ER-alpha for DNA stabilization (A), cell proliferation (B), and fuel supply (C). Circuit A: Estrogen- (*E*_2_) activated estrogen receptor alpha (ER*α*) upregulates estrogen signaling via a regulatory circuit together with genome stabilizer protein (BRCA1) and aromatase enzyme (A450). Activated ER-alpha induces messenger RNA (mRNA) expressions on *ESR1, BRCA1*, and *Cyp19A* aromatase promoter regions upregulating the synthesis of ER-alpha, BRCA1, and aromatase enzyme. Aromatase enzyme produces estrogen hormones for further ER activation. In addition, activated ER-alpha may induce activating mutations on *ESR1, BRCA1*, and *Cyp19A* genes through the expression and activation of appropriate long noncoding RNAs (lncRNAs). In addition, ER-alpha and BRCA proteins are capable of direct binding as transcriptional factors regulating each other's activity. Circuit B: Estrogen activated ER*α* is the crucial regulator of increased and decreased cell proliferation in strong interplay with membrane-associated tyrosine kinase growth factor receptors, EGFRs and IGF-1Rs. ERs regulate the expression and activation of growth factors (GFs) and their receptors. Transduction of growth factor signaling (GFS) induces kinase cascades via PI3-K/AKT/mTOR and RAS/RAF/MEK/MAPK pathways, which are transmitted into the nucleus inducing expressions of specific genes through an unliganded activation of ERs. Circuit C: Estrogen activated ER*α* is the regulator of all steps of cellular glucose uptake and the maintenance of glucose homeostasis. Estrogen-regulated genes stimulate both insulin synthesis and insulin receptor (IR) expression. Activated ER*α* stimulates the expression and activation of GLUT4 facilitating cellular glucose uptake. In addition, estrogen-activated ER*α* at the plasma membrane stimulates the kinase cascade of the PI3-K/AKT/mTOR pathway via IRS-1 activation. These signals induce specific gene expressions in the nucleus conferred by unliganded ER*α* activation. CA: coactivator, AF2: ligand binding domain, and AF1: nonligand binding domain.

Estrogen-activated ER-alphas are the primary initiators and organizers of the regulatory circuit for DNA stabilization in a triangular partnership with genome safeguarding proteins, such as BRCA1 and aromatase enzyme (A450) (Figure 1; circuit A). The promoter regions of *ESR1*, *BRCA1*, and *CYP19* aromatase genes exhibit a strong interplay for the appropriate expression of ER-alpha, BRCA1 protein, and aromatase enzyme [32]. Upregulation of ER signaling is the prerequisite of safe cell proliferation in both amplifying and quenching phases.

Activated ER*-*alpha drives the transcriptional activity on *ESR1* gene inducing high expressions of protein coding ER-alpha mRNAs and leading to a self-generating overexpression of ER-alpha protein. Activated ER-alphas also have the capacity to occupy *BRCA1* promoter regions increasing the expression of protein coding BRCA1 mRNA transcripts and a consequentially elevated BRCA1 protein synthesis [46]. In healthy cells, there are no reports on the capacity of activated ER-alpha to occupy the *CYP19 A* promoter region and to induce directly an increased aromatase enzyme expression [44]. Conversely, in breast cancer cells lines, estradiol treatment induces a rapid increase in aromatase expression and estrogen synthesis by unliganded activations of ER-alpha via growth factor-mediated pathways [47, 48].

Abundant, activated ER-alphas may drive activating mutations on various genes conferred by its lncRNA transcripts, including HOTAIR [44, 49]. Highly expressed lncRNAs provoke amplification on *ESR1* gene leading to further overexpression and an increased estrogen-binding capacity of ERs [47]. Abundant lncRNA transcripts of ERs are capable of inducing activating mutations on *BRCA1* gene as well, leading to its amplification and a consequential abundant BRCA1 protein expression [50, 51].

BRCA1 protein as a transcriptional factor increases the transcriptional activity of *BRCA1* gene and induces an abundant expression of newly formed BRCA1 protein [32]. BRCA1 protein activates the expression of *ESR1* gene and a consequentially increased ER-alpha synthesis [52]. In addition, BRCA protein may occupy the *CYP19A* promoter region, which is BRCA1 responsive and confers an increased expression of aromatase enzyme. BRCA1 protein ensures a safety balance between the expression of ER-alpha protein and aromatase enzyme [44].

Abundant BRCA1 proteins may induce activating mutations on *ESR1*, *BRCA1*, and *CYP19* aromatase genes [44]. BRCA1 occupies lncRNA promoters increasing lncRNA expression. Appropriate lncRNAs may provoke activating mutation and amplification on *ESR1* gene promoting increased expression and activation of ER-alpha. Moreover, a BRCA1 protein-stimulated expression of certain lncRNA transcripts confers activating mutations and amplification of *BRCA1* genes as well, inducing an enhanced expression of BRCA1 protein. Increased expression of BRCA1 protein upregulates the transcriptional activity of ER-alpha conferred by either cyclin D1 [53] or p300 coactivator protein [54]. An increased BRCA1 activity mediates a repressed unliganded activation of ERs [55], while a compensatory increase in liganded ER activation strongly improves DNA stability [39]. Furthermore, lncRNA transcripts of BRCA1 may stimulate amplification on *CYP19* aromatase promoter gene, and consequentially increased A450 aromatase enzyme synthesis leading to an abundant conversion of androgens to estrogens [56, 57]. Increased estrogen concentrations bind and activate abundant ER-alphas, further stimulating the upregulative circuit of DNA stabilization [32].

In *BRCA1* mutation carriers, the liganded ER activation is repressed, while the mutant BRCA1 protein drives high aromatase levels and compensatory increase in estrogen synthesis through the selection of the appropriate *CYP19* aromatase promoter region [57].

BRCA1 and ER-alpha proteins are capable of direct binding as well, as transcriptional factors. Certain binding sites drive upregulative processes, while others may silence the transcriptional activity [58]. Mutagenic alteration or low expression of ER-alpha dangerously decreases the expression of BRCA1 mRNA transcripts and BRCA1 protein synthesis, weakening DNA safeguarding [59]. In turn, the decreased or defective synthesis of BRCA1 protein leads to downregulation of both ER-alpha mRNA expression and ER-alpha protein synthesis [60]. A downregulative interaction between ER-alpha and BRCA1 protein results in an unrestrained proliferation of tumor cells [32].

### 2.3. Cell Proliferation Circuit Regulated by Estrogen-Activated ER-Alpha

The main regulator of cell proliferation is the estrogen-activated ER-alpha in strong interplay with membrane-associated tyrosine kinase growth factors receptors, such as the epidermal growth factor receptor (EGFR) and insulin-like growth factor receptor 1 (IGF-1R) (Figure 1; circuit B). ER-alpha activation ensures a strong control over DNA replication during both increased and decreased cell proliferation [32]. Close upregulative crosstalk between ER and growth factor receptor (GFR) signaling ensures safety DNA repair and stability during all phases of growth factor-mediated cellular processes [39].

IGF-1R exhibits a bidirectional signaling pathway with estrogen-activated ERs [61]. IGF-I expression is regulated by insulin and growth hormone (GH), which stimulate the synthesis of IGF-I in the liver, the main source of circulating IGF-I [62]. IGF-1 binding to IGF-1R activates two main signaling pathways: the phosphatidylinositol 3-kinase (PI3K)-AKT) and the Ras-mitogen-activated protein kinase (MAPK) pathways. These kinase cascades stimulate an unliganded transcriptional activity of ER-alpha via phosphorylation of serine residues [63].

ERs are capable of stimulating many proteins in the insulin-IGF-1 system, including IGF-1R and insulin receptor substrate 1 (IRS-1) [64, 65]. ER-alpha binds and phosphorylates IGF-1R and controls its signaling pathways, while in IGF-1 knock-out mice, estradiol-induced uterine growth is missing [66]. In turn, in vivo IGF-1 activation of uterine cell proliferation is strongly dependent on the ER-alpha activity [67].

Estrogen treatment stimulates the synthesis of EGF in uterine epithelium via ER activation leading to a strong proliferative effect [68]. In the absence of estrogen, EGFR signaling may be dependent on unliganded ER activation [69]. In the uterus of ER-alpha knock-out mice, EGF induced DNA synthesis, and transcription was completely missing [38]. In ovariectomized mice, 17-beta-estradiol treatment caused a rapid transient upregulation of uterine EGFR mRNA and protein levels and increased the number of EGF-binding sites through ER activation [70].

In the nucleus, EGFR signal is capable of phosphorylation and activation of ER-alpha at serine 118 through the growth factor receptor-activated MAPK pathway [71, 72]. Phosphorylation at serine 118 increases ER-related transactivation of several genes that are upregulated by EGFR. Growth factor receptor signal may also increase the transcriptional activity of nuclear ERs via the phosphorylation of their coactivator proteins, including steroid receptor coactivator 1 and p300 protein, as well as through interaction with cyclin D1 [73, 74].

Cytoplasmic, estrogen-activated ERs induce an upregulation of the PI3K signaling pathway via EGFR activation [75]. In endothelial cells, PI3K activation by estrogen treatment led to a rapid upregulation of 250 estrogen-regulated genes within 40 minutes [76]. The ER/EGFR crosstalk at the membrane ensures the activation of multiple signaling pathways that provides a profound increasing impact for the extensive transcriptional activity of ERs [61].

In human breast cancer, the expression of ERs and EGFRs exhibit an inverse correlation [77, 78]. In tamoxifen-responsive breast cancer cell lines, a compensatory increased expression of ERs may be observed. In tumors, becoming tamoxifen resistant, an additional high expression of growth factor receptors may be observed [23]. Abundant GFRs extremely increase the unliganded activation of ERs as a counteraction to the artificial blockade of AF2 domain, while it is incapable of restoring DNA stability when the liganded activation is continuously blocked.

### 2.4. Fuel Supply Circuit Regulated by Estrogen-Activated ER-Alpha

Estrogen-activated ER-alpha drives a regulatory circuit for the maintenance of glucose homeostasis and upregulates all steps of cellular glucose uptake providing fuel for cellular mechanisms (Figure 1, circuit C.). Defects of ER signaling lead to serious difficulties in cellular glucose uptake designated as insulin resistance and result in serious chronic diseases including cancer [32].

Estrogen-regulated genes stimulate insulin secretion, insulin receptor expression, and activation [79]. When insulin binds to the insulin receptor, autophosphorylation of multiple tyrosines initiates the activation of insulin signal transduction [80]. Activated ERs may upregulate the expression and functional activity of the intracellular glucose transporter-4 (GLUT4) facilitating insulin-assisted glucose uptake [81]. ER-alpha regulates the insulin receptor substrate 1- (IRS1-) mediated activation of the PI3-K/mTOR signaling pathway and receives a feedback signal through the unliganded activation of its AF1 domain [82].

In MCF-7 human breast cancer cell line, estradiol has potentiating effects on insulin signaling via increasing the expression of the insulin receptor substrate-1 (IRS-1) [83]. In ZR-75-1 breast cancer cells, glucose transporter 1 (GLUT1) expression was upregulated by a combined estrogen/progesterone treatment [84]. In MCF-7 cell lines, estradiol treatment-activated ERs upregulate the PI3K/Akt signaling pathway parallel with a facilitated translocation of glucose transporter 4 (GLUT4) vesicles to the plasma membrane [85]. These results reveal the mechanisms through which estrogen improves insulin-assisted glucose uptake even in cancer cells, providing energy for the restoration of DNA stability [32].

## 3. Correlations among Defects of Estrogen Signaling, Breast Cancer Risk, ER Expression, and the *ESR1* Status of Tumors

All well-known cancer risk factors are in correlation with defects of estrogen signaling in partnership with glucose intolerance as estrogen regulates all steps of cellular glucose uptake [86, 87]. Either estrogen deficiency or a defective ER activation means high risk for breast cancer when the compensatory mechanisms are insufficient [36].

Estrogen deficiency in the perimenopausal phase (45–55 yrs) of women is a high risk for breast cancer when the compensatory peripheral synthesis of estrogens is delayed. The insufficient estrogen supply induces an increasing expression of mammary ERs. Breast cancers, initiated in the estrogen deficient perimenopausal period, are typically strong ER-positive tumors [87].

Defective liganded ER activation is a high breast cancer risk for germline *BRCA1/2* gene mutation carrier women [32]. In *BRCA1* deficient human ovarian cancer cells, the liganded transcriptional activity of ERs showed a relative decrease [58], while ER-alpha exhibited a compensatory-increased unliganded transcriptional activity associated with *BRCA* mutation [64]. In *BRCA* mutation carriers, the failure of liganded ER activation is associated with low ER expression levels, and they show an increased inclination to the development of poorly differentiated ER-negative breast cancers [87].

Visceral obesity is a high-risk for overall breast cancer in both young and postmenopausal women as it is strongly associated with a deficient estrogen signaling and defective glucose uptake [35, 45]. Circumference measures of abdominal fat mass (waist, hip, and waist/hip ratio) were linearly associated with increased risks for ER-negative and triple-negative breast cancers (TNBCs) in both premenopausal and postmenopausal women [88, 89]. In conclusion, obesity-associated defective estrogen signaling allows easier survival for ER-negative tumors than for ER-positive ones, resulting in a relative accumulation of ER-negative and TNBC-type tumors among patients with central obesity [87].

Insulin resistant states, such as metabolic syndrome and type-2 diabetes, are strong risk factors for breast cancer in women. It is a widely accepted fact that the higher the grade of insulin resistance in women with or without obesity, the higher is the risk for breast cancer, particularly for more aggressive ER-negative types [90].

Genetically defined anovulatory infertility in young women is a preferential cancer risk for the female organs having high estrogen demand: breast, endometrium, and ovaries [35]. Among infertile women with weak estrogen signaling, a relatively increased prevalence of poorly differentiated ER-negative breast tumors is a characteristic feature [87]. Genetic defects of *CYP19 A* gene may induce aromatase deficiency and low estrogen levels, while an inherited ESR1 gene mutation is frequently associated with ER resistance and compensatory elevated estrogen levels [39]. These observations underline the fact that a weak estrogen signaling may be associated with increased breast cancer risk independent of either low or high serum estrogen concentrations.

In healthy cells, the *ESR1* gene copy number and ER expression exhibit a direct correlation; consequently, an amplification of *ESR1* gene is physiologically associated with an increased ER expression. In breast cancers, correlations between *ESR1* gene status and estrogen receptor protein levels were measured by ligand binding assay (LBA) and immunohistochemistry [91]. *ESR1* amplification was the most frequent among tumors exhibiting more than 75% ER-positive cells (35%) or showing a high value of LBA (33%). Among breast cancers classified as ER-negative ones, a large fraction exhibited *ESR1* deletion (55%).

Correlations between *ESR1* amplification in tumors and improved survival of patients were reported in two studies [92, 93], whilst a third study reported a poor survival of patients with tumors harvesting an increased *ESR1* copy number [94]. These ambiguous findings may be explained with the heterogeneous regulatory disturbances in tumors; similar *ESR1* amplification values may be coupled with various levels of ER content and immune reactivity [91]. *ESR1* amplification was observed even in certain ER-negative tumors, while it was associated with a poor survival of the patients [95]. Recently, a close association between the DNA repair capacity (DRC) and the ER expression status of breast cancers was reported [96]. These data suggest that the ER status of tumors shows closer correlation with a good prognosis of the disease as compared with *ESR1* amplification.

## 4. Molecular Events behind Estrogen Treatment-Induced Responses of Breast Cancers

In ER-positive tumor cells, estrogen treatment results in a strong upregulation of ER signaling initiating a restoration of the circuit of DNA stabilization and inducing a consequential programmed cell death [32]. In ER-negative breast cancer cells, estrogen treatment provoked tumor response after inoculation of exogenous ERs [97].

In tumor cells lines, estradiol treatment stimulates both liganded and unliganded ER activations. In ER-positive breast cancer cell lines, estrogen treatment increases the expression and transcriptional activity of ERs [98]. In breast cancer cells, estrogen treatment increased the expression of EGFR and HER2 [23] and activated their signaling pathways upregulating even the unliganded activation of ERs. In tumor cells treated with estrogen, overexpression of ERs and GFRs ensures DNA restoration via liganded and unliganded ER activation [39].

In breast cancer cells, estrogen administration usually induces an amplification of *ESR1* gene at 6q25 locus upregulating ER protein synthesis [92]. During breast cancer “adaptation” to estrogen, a cluster of noncoding RNAs was observed activating the *ESR1* locus [47]. Patients exhibiting *ESR1* gene amplification in their breast tumors experienced a longer disease-free survival as compared with those without it [93].

In tumor cells treated with estradiol, activated ERs mediate an increased expression of lncRNAs, including HOTAIR [44, 49]. Increased HOTAIR expression in the tumors of breast cancer cases was associated with markedly lower risks for relapse and mortality [99].

In breast cancer cells, estradiol treatment-activated ER-alpha increases the expression of aromatase enzyme via an increased lncRNA transcription and a mutation of *CYP19A* gene. Estradiol-activated ERs upregulate the aromatase activity as well by means of enhanced tyrosine phosphorylation [48]. Estradiol treatment elevated the aromatase activity in a dose-dependent manner even in ER-negative tumor cells when they were transfected with exogenous ER-alpha protein [100]. In breast cancer cases, a direct correlation was experienced between the aromatase activity of removed tumor samples and patient's survival time after surgery [101].

In MCF-7 tumor cell line, estrogen treatment induced an increased expression of BRCA1 protein [50]. In turn, BRCA1 protein induces ES*R1* gene amplification resulting in an upregulation of ER protein synthesis in breast cancer cell lines [52]. These observations justify that estrogen-activated ER-alpha and BRCA1 protein work in close partnership in the upregulation of DNA stabilizer circuit.

## 5. Molecular Events behind the Response and Resistance of Breast Cancer Cells under Antiestrogen Treatment

**Figure 2:**
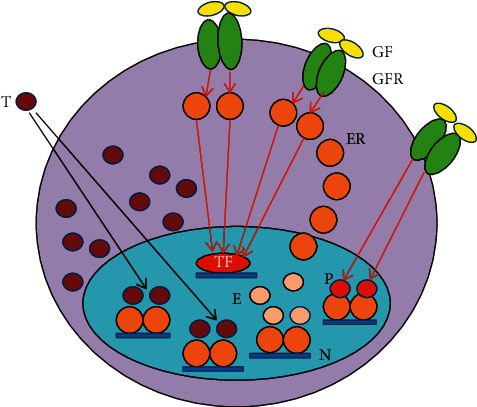
Emergency response to tamoxifen (T) treatment in tumor cells. The rapid translocation of unbound estrogen receptors (ERs) out of the nucleus facilitates their interactions with membrane-associated growth factor receptors (IGF1-R and EGFR) inducing their unliganded activation. Activated cytoplasmic ERs initiate rapid transcriptional processes in the nucleus through transcriptional factors (TFs). Growth factor- (GF-) activated GFRs may also induce unliganded activation on nuclear unbound ERs driving their transcriptional activity. E: estrogen; P: phosphorylation; N: nucleus; red arrow: activation; black arrow: inhibition.

**Figure 3:**
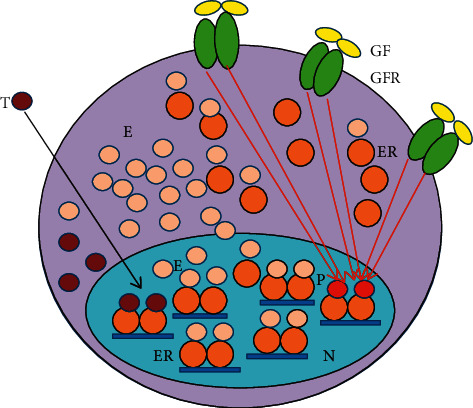
Mechanism of tumor response in cancer cells treated with tamoxifen (T). Abundant unliganded estrogen receptor (ER) activation increases the expression of estrogen-regulated genes upregulating the circuit of ER-aromatase-E2-ER expression. In the meantime, growth factors (GFs) activate growth factor receptors (GFRs) activating free nuclear ERs via an unliganded pathway. The predominance of estrogen- (E-) bound ERs over T-bound ones leads to DNA repair, apoptotic death, and clinical tumor response. P: phosphorylation; N: nucleus; red arrow: activation; black arrow inhibition.

**Figure 4:**
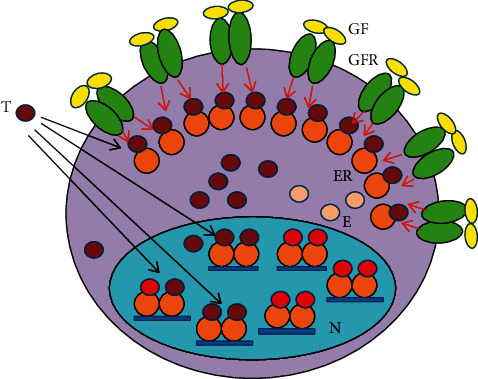
Mechanism of tumor resistance in cancer cells treated with tamoxifen (T). The liganded activation of abundant estrogen receptors (ERs) is completely blocked by T binding, and they are deregulated instead of activation. Compensatory abundant expression of growth factor receptors (GFRs) struggles for the unliganded activation of T-bound ERs, while the T blockade of the liganded pathway inhibits the restoration of ER signaling. GF: growth factor; N: nucleus; red arrow: activation; black arrow: inhibition.

In breast cancer cells, there are slight, moderate, or serious errors in the pathways of the genome stabilizer circuit, resulting in different grades of differentiation and different failures in the regulatory processes. In case of newly diagnosed breast tumors, the stronger the ER signaling, the better is the expectable prognosis of the disease [87]. In ER-positive antiestrogen-responsive tumors, the medical blockade of liganded ER activation is compensated via increased estrogen synthesis and ER expression upregulating ER signaling (Figures 2 and 3.) In antiestrogen resistant tumors, activating mutations help the increased expression and activation of GFRs so as to increase the compensatory, unliganded activation of ERs. The abundant expression of growth factor receptors (GFRs) struggles for the unliganded activation of blocked ERs, while the blockade of the liganded pathway inhibits the restoration of ER signaling (Figure 4.)

### 5.1. Molecular Events in Antiestrogen-Responsive Breast Cancers

In antiestrogen-responsive tumors, the principal action against the blockade of AF2 domain is a compensatory restoration and upregulation of liganded ER activation [44].

(1) In breast cancer cells, tamoxifen treatment facilitates the translocation of ER-alphas out of the nucleus and enhances their interaction with membrane-associated EGFRs, leading to a prompt compensatory unliganded activation of ERs [21] (Figure 2.). (2) In human breast cancer cells, long-term estrogen deprival upregulates the expression of the most studied coactivator of ER-alpha, AIB1 (amplified in breast cancer 1) [102]. Tamoxifen induces a redistribution of cyclin D1 from STAT3 to the ER, which increases the activation of both STAT3 and ERs [103]. (3) In tumors treated with tamoxifen, an excessive activation of the transcription factor, NF*κ*B, and its upregulative crosstalk with ER-alpha was documented [104, 105]. (4) In tumors, tamoxifen provokes an increasing expression of certain microRNAs so as to bind to mRNA transcripts of ERs, activating the translational processes facilitating new protein synthesis [42]. (5) In tamoxifen-responsive tumors, the increased copy number of *ESR1* is typically coupled with an increased expression and activation of ERs [92, 93] (Figure 3). (6) AI treatment in tumor cells induces an acquired amplification of aromatase encoding *CYP19A1* gene enhancing both enzyme expression and estrogen synthesis [106]. (7) In antiestrogen-treated tumor cells, copious lncRNA transcripts of ERs confer activating mutations for crucial genes participating in the genome stabilizer circuit, such as *ESR1, BRCA1*, and *CYP19A* [44].

In breast cancers responsive to antiestrogen treatment, the facilitated regulatory processes may lead to successful tumor response when the compensatory restoration of liganded ER activation may keep up with the continuous artificial blockade of estrogen signaling [107].

### 5.2. Molecular Events in Breast Cancers Becoming Unresponsive to Antiestrogen Treatment

When an earlier antiestrogen-responsive breast cancer exhausted the possibilities for liganded ER activation, the upregulation of unliganded ER activation through the growth factor receptor signal remains as an ultimate refuge for DNA stabilization [39]. However, even an extreme increase in unliganded ER activation is incapable of restoring ER signaling when the liganded pathway is completely blocked (Figure 4).

There are physiological pathways for increasing unliganded ER activations. In tamoxifen-resistant tumor cells, an increased expression of the ER coactivator HOXB7 induces an enhanced kinase domain phosphorylation of both EGFR [108] and HER2 [109] increasing the unliganded activation of ERs. In endocrine-resistant breast cancer, the estrogen receptor coactivator AIB1 and HER2/neu signaling stimulates hormone-independent ER activation [110]. In breast cancer xenografts, an acquired resistance to endocrine and HER2-targeted therapies is associated with the compensatory upregulation of MUCIN4 [111]. In tamoxifen-resistant breast cancers, the upregulation of the transcriptional factor NF*κ*B highly activates ERs [112]. In endocrine-resistant tumors, an increased expression of either EGFRs [113] or IGF-1 Rs [114] at the plasma membrane amplifies the unliganded activation of ERs. In endocrine-resistant cancers, an elevated IGF-1R signaling increases unliganded ER activation through the MAPK/ERK and PI3K/Akt pathways [115]. In tamoxifen-resistant breast cancer, IGF-1R signaling supports the upregulation of EGFR conferring together an increased unliganded activation of ERs [116].

In endocrine resistant tumors, acquired mutations may extremely increase unliganded ER activation. (1) In tumors resistant to tamoxifen, ER-mediated activating mutation of *ERBB2* gene of growth factor receptor tyrosine kinases increases the expression and activity of growth factor receptors conferring unliganded activation for ERs [110]. (2) In endocrine refractory ER-positive breast cancer, *PIK3CA* gene is frequently mutated upregulating the components of the PI3K-AKT-mTOR pathway and increasing the unliganded activation of ERs [117]. (3) Breast cancers resistant to AIs frequently exhibit acquired point mutations in the ligand binding domain (LBD) of *ESR1* gene conferring a constitutive hormone-independent activation of ERs [118]. (4) In antiestrogen-resistant tumors, chromosomal rearrangement affecting *ESR1* gene is a further mutational mechanism driving an increased unliganded activation of ERs [24]. (5) In tamoxifen-resistant breast cancer cells, a highly activated PI3K/AKT pathway is associated with the significant upregulation of BARD1 and BRCA1 protein expressions through an increased unliganded activation of ERs [119].

### 5.3. How Can Estrogen Achieve an Apoptotic Death in Endocrine Resistant Tumors?

Estrogen treatment of breast cancers resistant to either long-term estrogen deprivation (LTED-R) or tamoxifen (TAM-R) triggers an apoptotic death in tumors [120, 121]. Considering the strong upregulation of both ER and GFR expressions in breast cancers unresponsive to antiestrogen treatment, estrogen is capable of exerting its physiological anticancer capacity via a balanced liganded and unliganded activation of abundant ERs. In reality, estrogen does not restore the “antiestrogen sensitivity” of unresponsive breast cancer, but it rather helps tumor cells to get rid of the poisonous medicament.

In raloxifene-resistant MCF-7 tumors, the continuous endocrine treatment stimulated ER expression and tumor growth, while a 17-beta estradiol treatment induced an apoptotic action and significantly reduced tumor sizes [122]. In the background, estradiol binding utilized the abundant expression of ERs and GFRs restoring a balanced liganded/unliganded ER activation and promoting an apoptotic response [39]. In tamoxifen-stimulated MCF-7 tumors, estradiol treatment induced a striking apoptotic activity, while a parallel fulvestrant treatment stimulated again the growth of tumors via disturbing the ER-alpha-mediated regulation of Fas, HER2/neu, and NF*κ*B [123]. In a tamoxifen-stimulated tumor model, estradiol-induced tumor regression required activation of ER-alpha, FasL/FasL ligand, and Akt pathways [124].

Long-term estrogen deprivation (LTED) highly increased ER expression in an MCF-7 cell line associated with a rapid hormone-independent growth [18]. In this LTED model, even extremely low estradiol concentrations (<10^−11^M) were capable of initiating an apoptotic death. The authors suggested a mitochondrial pathway having a crucial role in estradiol-induced apoptosis. In LTED breast cancer cells, adenosine monophosphate-activated protein kinase (AMPK) was an important mediator of estradiol-induced apoptosis [125]. In tumors resistant to LTED, estradiol-induced ER activation upregulated proinflammatory genes including IL, IFN, and arachidonic acid-related genes [126]. In LTED breast cancer cells, activation of glucocorticoid receptors blocked the estradiol-induced apoptosis via the suppressed expression and transcriptional activity of NF*κ*B [127].

## 6. Conclusion

Cancer cells are embarrassed human cells exhibiting remnants of the same mechanisms for DNA stabilization like patients have in their healthy cells. Antiestrogens are chemotherapeutic agents targeting the liganded activation of ERs, which is the principal means of genomic regulation in both patients and their tumors.

Antiestrogen treatment is a triple attack targeting the genomic machinery of the tumor, the adjacent breast tissue, and the whole body of patient. Antiestrogen treatment of advanced breast cancers may result in a primary tumor response in the minority of cases; however, doctors may abandon the therapy in a hurry when tumors show stagnation or turn to a paradox growth.

In contrast, the wide spread use of postsurgical, prophylactic antiestrogen treatment for breast cancer cases is a blind risk-taking. It is quite impossible to predict that when a long-term antihormone treatment will turn to an uncompensated phase. With the exhaustion of compensatory processes, breast cancer will return and grow. In addition, the adjacent deregulated breast tissue becomes incapable of tumor demarcation, and deregulated remote organs cannot defend themselves from the colonization of copiously arriving malignant cells. These processes delineate the mechanism of local recurrences and metastatic spread of tumors in breast cancer cases, in strong correlation with long-term antiestrogen treatment.

In antiestrogen-responsive tumor cell lines, compensatory increased expression and liganded activation of estrogen receptors (ERs) lead to apoptotic death. Conversely, in antiestrogen resistant tumors with exhausted liganded ER activation, a compensatory increased unliganded ER activation may be experienced, conferred by the upregulation of growth factor receptor signals and acquired activating mutations. Researchers mistakenly suspect that tumor cells resistant to antiestrogen therapy fight for their survival via activating their ERs through hormone-independent pathways.

As a next step, the pharmaceutical industry worked on the development of compounds targeting the signaling pathways of tyrosine kinase growth factor receptors so as to achieve an additional inhibition of the unliganded activation of ERs. Nevertheless, in breast cancer cases, the double blockade of ER activation resulted in doubtful tumor responses and severe toxic complications.

From the early 2000s, apoptotic effects of estrogen treatment on breast cancer cell lines resistant to either tamoxifen or estrogen withdrawal have been investigated. This striking experience suggested that estrogen is capable of returning antiestrogen resistant tumors to antiestrogen-responsive ones. In the past two decades, scientists have been working on answering two highly exiting questions. First: how can antiestrogens be frequently ineffective in breast cancer care, while it is “well-known” that estrogen drives cancer development? Second: how can estrogens be highly effective anticancer agents even against tumors growing under exhaustive antiestrogen treatment when estrogens are “well-known” promoters of unrestrained tumor proliferation?

The 50-year period of antiestrogen therapy and the study of genomic mechanisms in antiestrogen-responsive and antiestrogen-unresponsive breast cancers have considerably enhanced our understanding regarding estrogen signaling. The impressive capacity of liganded ERs for genome modification in emergency states provides excellent possibilities for estrogen/ER use in all fields of medical practice including cancer therapy.
